# Platelet-Released Growth Factors Induce Genes Involved in Extracellular Matrix Formation in Human Fibroblasts

**DOI:** 10.3390/ijms221910536

**Published:** 2021-09-29

**Authors:** Andreas Bayer, Bernard Wijaya, Franziska Rademacher, Lena Möbus, Mark Preuß, Michael Singh, Mersedeh Tohidnezhad, Yusuke Kubo, Meno Rodewald, Peter Behrendt, Jan-Tobias Weitkamp, Regine Gläser, Jürgen Harder

**Affiliations:** 1 Institute of Anatomy, Kiel University, 24098 Kiel, Germany; m.singh@anat.uni-kiel.de; 2 Department of Dermatology, University Medical Center of Schleswig-Holstein, Campus Kiel, 24105 Kiel, Germany; wjybernard@gmail.com (B.W.); frademacher@dermatology.uni-kiel.de (F.R.); lmoebus@dermatology.uni-kiel.de (L.M.); stu104302@mail.uni-kiel.de (M.R.); 3 Department for Vascular Medicine, University Heart and Vascular Center Hamburg, University Medical Center Hamburg-Eppendorf, 20246 Hamburg, Germany; ma.preuss@uke.de (M.P.); rglaeser@dermatology.uni-kiel.de (R.G.); jharder@dermatology.uni-kiel.de (J.H.); 4 Department of Anatomy and Cell Biology, RWTH Aachen University, 52074 Aachen, Germany; mtohidnezhad@ukaachen.de (M.T.); ykubo@ukaachen.de (Y.K.); 5 Department of Trauma Surgery, University Medical Center of Schleswig-Holstein, Campus Kiel, 24105 Kiel, Germany; peter.behrendt@uksh.de; 6 Department of Oral and Maxillofacial Surgery, University Medical Center Schleswig-Holstein, Campus Kiel, 24015 Kiel, Germany; jan-tobias.weitkamp@uksh.de

**Keywords:** platelet-released growth factors (PRGF), wound healing, extracellular matrix (ECM), fibroblasts

## Abstract

Platelet concentrate products are increasingly used in many medical disciplines due to their regenerative properties. As they contain a variety of chemokines, cytokines, and growth factors, they are used to support the healing of chronic or complicated wounds. To date, underlying cellular mechanisms have been insufficiently investigated. Therefore, we analyzed the influence of Platelet-Released Growth Factors (PRGF) on human dermal fibroblasts. Whole transcriptome sequencing and gene ontology (GO) enrichment analysis of PRGF-treated fibroblasts revealed an induction of several genes involved in the formation of the extracellular matrix (ECM). Real-time PCR analyses of PRGF-treated fibroblasts and skin explants confirmed the induction of ECM-related genes, in particular transforming growth factor beta-induced protein (TGFBI), fibronectin 1 (FN1), matrix metalloproteinase-9 (MMP-9), transglutaminase 2 (TGM2), fermitin family member 1 (FERMT1), collagen type I alpha 1 (COL1A1), a disintegrin and metalloproteinase 19 (ADAM19), serpin family E member 1 (SERPINE1) and lysyl oxidase-like 3 (LOXL3). The induction of these genes was time-dependent and in part influenced by the epidermal growth factor receptor (EGFR). Moreover, PRGF induced migration and proliferation of the fibroblasts. Taken together, the observed effects of PRGF on human fibroblasts may contribute to the underlying mechanisms that support the beneficial wound-healing effects of thrombocyte concentrate products.

## 1. Introduction

Platelet concentrate products, such as Platelet-Rich Fibrin (PRF) or Platelet-released growth factors (PRGF), are increasingly used worldwide in many areas of regenerative medicine [1] because they contain a multitude of growth factors, cytokines, and chemokines [2]. In the context of wound healing, it has been shown that 70% of chronic or complicated wounds heal or become smaller under the treatment of PRF [3,4]. However, the underlying mechanisms for these positive wound healing effects under treatment with platelet concentrate products remain poorly understood. So far, we have shown that the treatment of human keratinocytes with PRGF and PRF leads to an induction of the antimicrobial peptides human beta-defensin-2 (hBD-2) [5], hBD-3 [6] and psoriasin [7] in keratinocytes and thus to a strengthening of the epithelial barrier function. Furthermore, we could demonstrate that the treatment of keratinocytes with PRGF leads to an accelerated differentiation in keratinocytes and thus keratinization of the skin [8]. In contrast, the proliferation of keratinocytes was inhibited by PRGF [9]. The beneficial effects of PRGF may also be attributed to its capacity to induce various factors in keratinocytes, which are essential for the formation of the extracellular matrix (ECM) during wound healing [10]. According to our previous results on keratinocytes, the aim of this study was to assess the influence of PRGF on human fibroblasts. To this end, we used whole transcriptome sequencing to get an overview of PRGF-regulated genes in human primary fibroblasts. As a result, we conclude that PRGF induces various ECM-associated factors in fibroblasts. Furthermore, the proliferation and migration of the fibroblasts were enhanced by PRGF.

## 2. Results

### 2.1. PRGF Mediates the Induction of ECM-Associated Factors in Human Primary Fibroblasts

**Figure 1:**
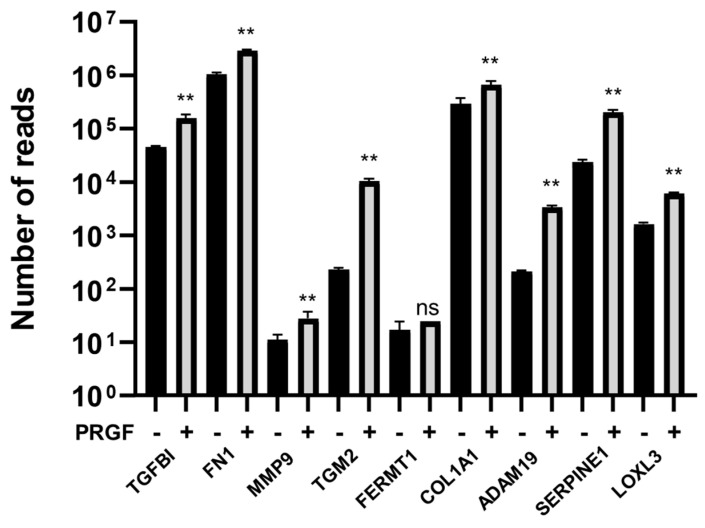
Whole transcriptome sequencing of PRGF-treated fibroblasts revealed induction of ECM-related genes. Human primary fibroblasts were treated with or without PRGF for 24 h. After stimulation, total RNA was isolated and used for whole transcriptome sequencing. Shown are means of the number of reads ± s.e.m. (*n* = 5, ** *p* < 0.01, ns = non-significant, Mann-Whitney *U* test).

To obtain an unbiased overview about the genes in fibroblasts that are regulated by PRGF, a whole transcriptome analysis was performed with fibroblasts stimulated with PRGFs derived from 5 different donors. This revealed a significant change in PRGF-mediated expression levels of 3664 genes (Table S1). A subsequent gene ontology (GO) enrichment analysis revealed the induction of various genes involved in the organization of the extracellular matrix. Specifically, transforming growth factor beta-induced protein (TGFBI), fibronectin 1 (FN1), matrix metalloproteinase-9 (MMP-9), transglutaminase 2 (TGM2), fermitin family member 1 (FERMT1), collagen type I alpha 1 (COL1A1), a disintegrin and metalloproteinase 19 (ADAM19), serpin family E member 1 (SERPINE1 or plasminogen activator inhibitor 1, PAI-1) and lysyl oxidase-like 3 (LOXL3) were induced by PRGF (Figure 1).

**Figure 2:**
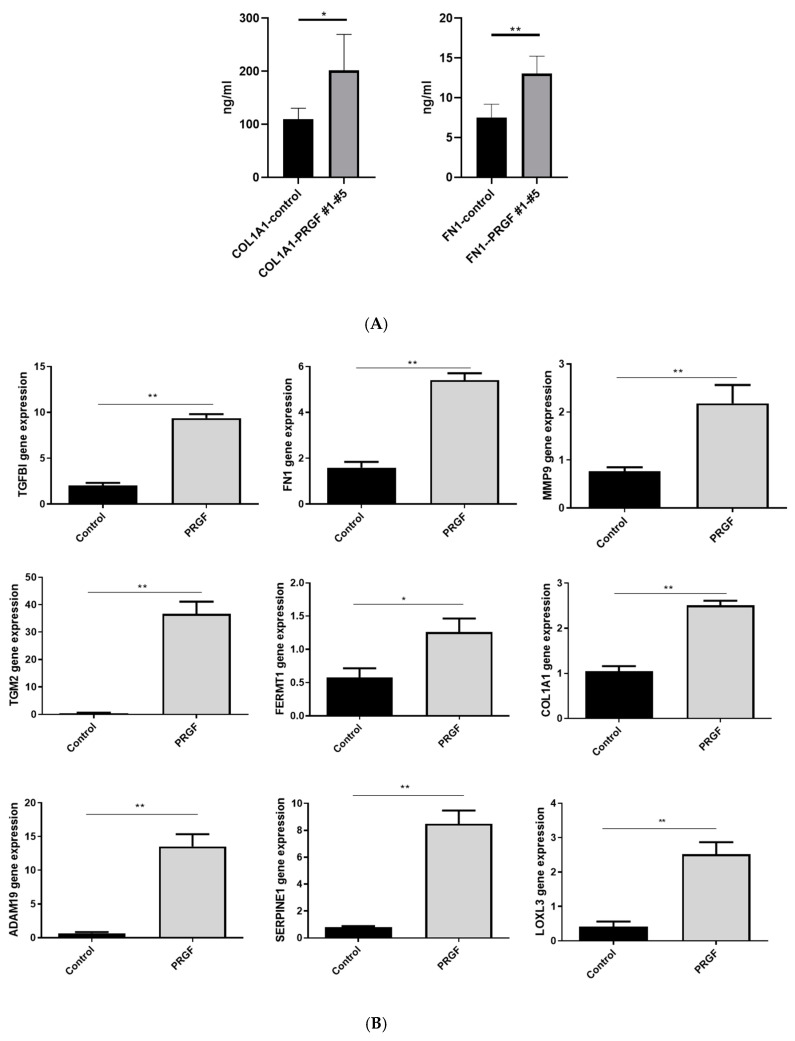

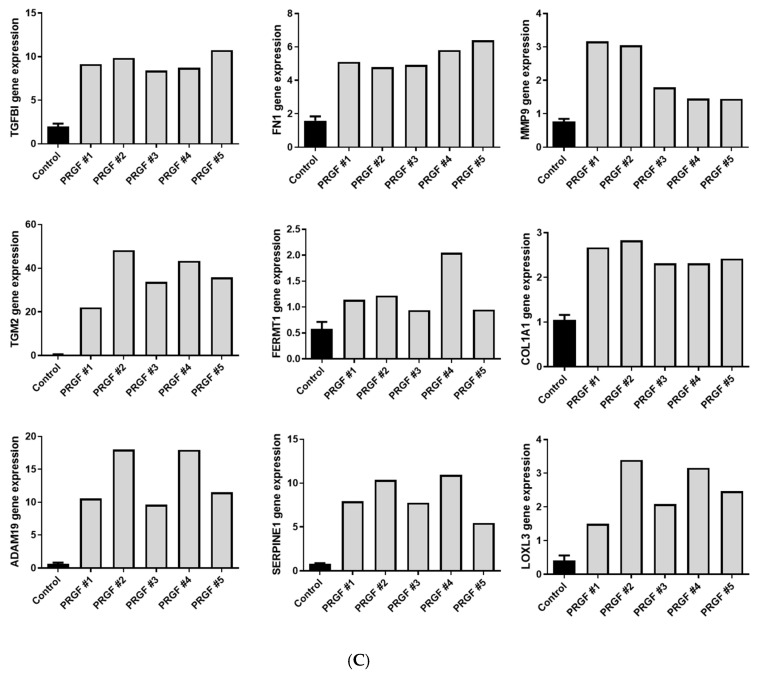
**PRGF induces expression of various ECM-related factors in human fibroblasts.** Human primary fibroblasts were stimulated for 24 h with PRGF (1:10) from 5 different donors (PRGF #1-PRGF #5). Relative gene expressions were determined by real-time PCR (**A**,**B**). Shown are induction levels of separate donors (**A**) or combined of all five different donors (**B**). Secretion of COL1A1 and FN1 was determined by ELISA (**C)**. Shown are means ± s.e.m. (*n* = 5, * *p* < 0.05, ** *p* < 0.01, Mann-Whitney *U* test).

Next, we used a real-time PCR to verify the PRGF-mediated induction of the genes identified by whole transcriptome sequencing. This confirmed the induction of all investigated genes in primary fibroblasts after 24 h of PRGF stimulation (Figure 2A,B). To determine whether gene induction resulted also in increased protein release, we measured protein concentration of fibronectin 1 (FN1) and the collagen type I alpha 1 (COL1A1) in the supernatants of the fibroblasts stimulated with five different PRGFs. This revealed an increased PRGF-mediated protein secretion (Figure 2C). 

### 2.2. The PRGF-Mediated Induction of ECM-Related Genes in PRGF-Treated Fibroblasts Is Time-Dependent

**Figure 3:**
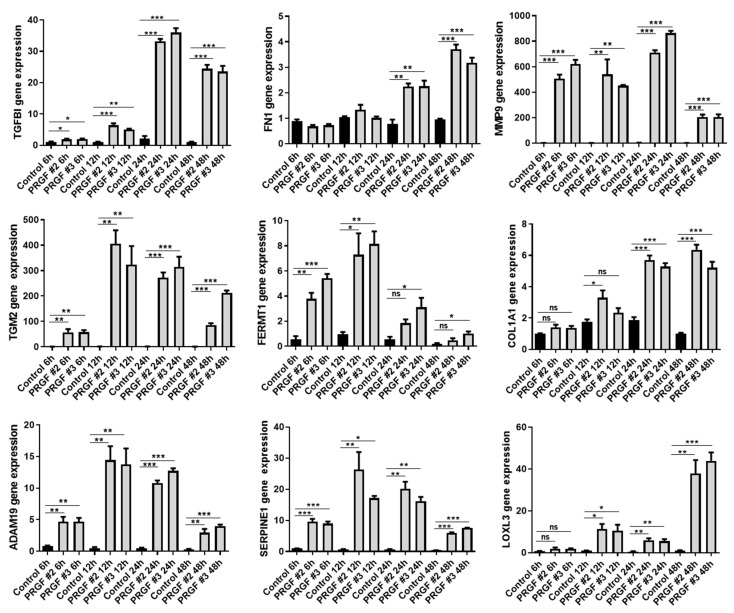
**Time kinetics of PRGF-induced ECM-related factors in human fibroblasts**. Human primary fibroblasts were stimulated with PRGF from two donors for the indicated periods. Relative gene expression was analyzed by real-time PCR. Shown are means ± s.e.m of three stimulations (* *p* < 0.05, ** *p* < 0.01, *** *p* < 0.001, ns = non-significant; ANOVA with Bonferroni’s multiple comparisons test).

A time kinetic study from 6 h to 48 h revealed a significant PRGF-mediated induction of all investigated genes (Figure 3). Except for FN1, all genes were induced already after 6 h of PRGF treatment. The PRGF-mediated induction of all genes persisted up to 48 h.

### 2.3. The PRGF-Mediated Induction of ECM-Related Factors in Human Fibroblasts Is Influenced by the Epidermal Growth Factor Receptor (EGFR)

**Figure 4:**
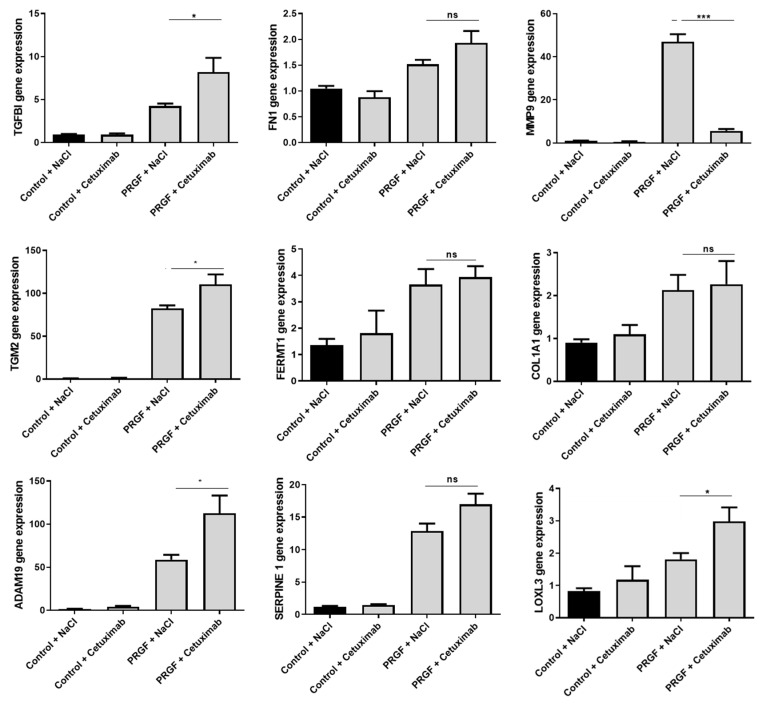
**The EGFR influences the PRGF-induced expression of TGFBI, MMP-9, TGM2, ADAM19 and LOXL3 in human fibroblasts.** Human primary fibroblasts were stimulated for 24 h with PRGF from a single donor in the presence or absence of the EGFR blocking antibody cetuximab. Relative gene expression was analyzed by real-time PCR. Shown are means ± s.e.m of three stimulations (* *p* < 0.05, *** *p* < 0.001; ns = non-significant, Student’s *t*-test).

In previous studies, we observed a relevant influence of the epidermal growth factor receptor (EGFR) on the PRGF-mediated induction of antimicrobial peptides and ECM-related factors in keratinocytes [5,6,7,10]. Therefore, in this study we aimed to analyze the influence of the EGFR on the observed PRGF-mediated induction of ECM-associated genes in fibroblasts. To this end, we used the monoclonal EGFR-antibody cetuximab to block and inactivate signal transduction by the EGFR. The blockade of the EGFR by cetuximab caused a significant inhibition of the PRGF-mediated gene induction of MMP9 in human fibroblasts. In contrast, treatment with cetuximab revealed a significant influence to enhance the PRGF-induced gene expression of TGFBI, TGM2, ADAM19 and LOXL3 (Figure 4).

### 2.4. PRGF Induces ECM-Related Factors in Ex Vivo Skin Explants

**Figure 5:**
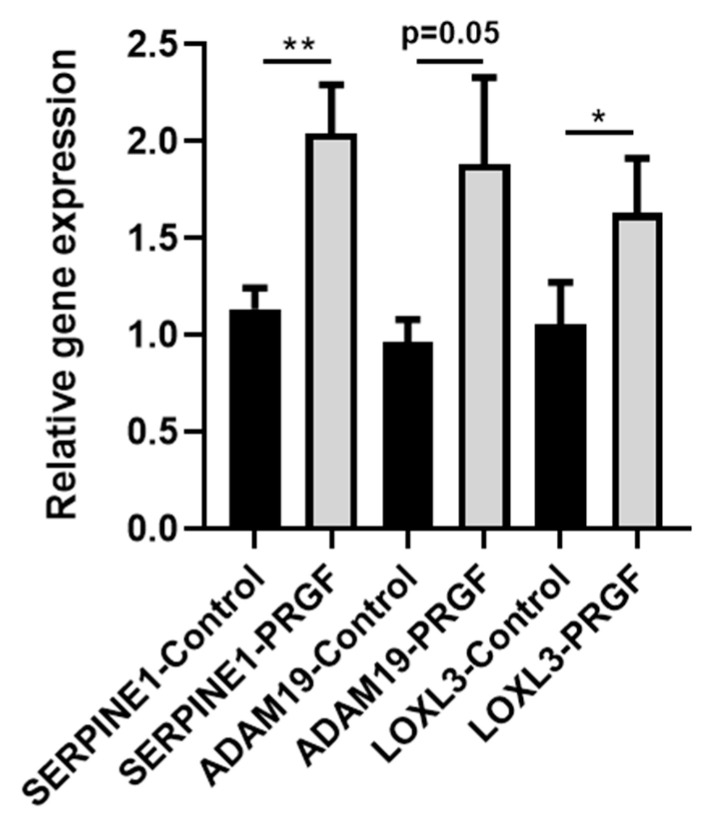
**PRGF induces expression of ECM-related genes in skin explants.** Human skin explants were stimulated with PRGF for 24. Gene expression was analyzed by real-time PCR. Shown are means ± s.e.m (*n* = 9–14; * *p* < 0.05, ** *p* < 0.01, Mann-Whitney *U* test).

To evaluate whether PRGF also induces ECM-related genes in total skin, we used skin explants derived from surgery and treated them with PRGF for 24 h. This revealed induction of SERPINE1, ADAM19 and LOXL3 gene expression (Figure 5). This aligns with our recent data showing induction of other ECM-related factors such as TGFBI, MMP9 and FERMT1 in ex vivo skin explants [10].

### 2.5. PRGF Treatment Induced Proliferation and Migration of Primary Human Fibroblasts

**Figure 6:**
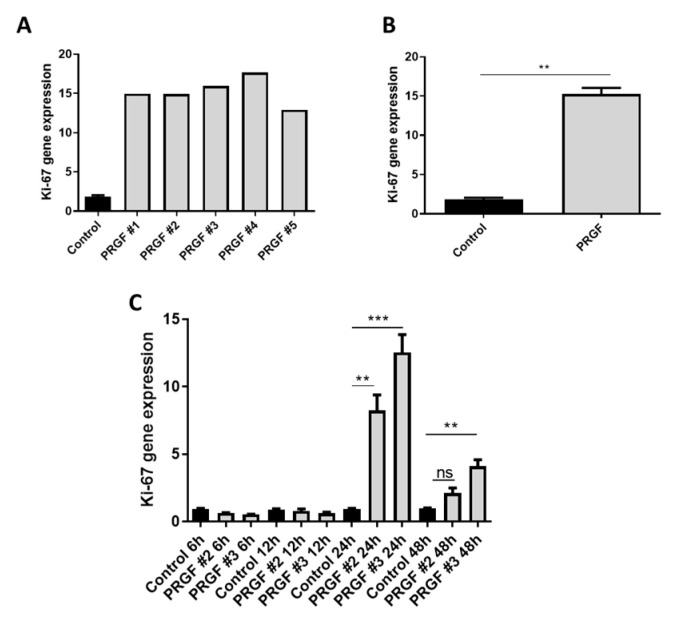
**PRGF treatment of primary human fibroblasts induced Ki-67 gene expression.** (**A**,**B**) Human primary fibroblasts were stimulated with PRGF from five different donors (PRGF #1-PRGF #5) for 24 h. Ki-67-gene expression was analyzed by real-time PCR. Shown are induction levels of separate donors (**A**) or combined of all 5 different donors (**B**) (*n* = 5; ** *p* < 0.01, Mann-Whitney *U* test). (**C**) Primary human fibroblasts were stimulated with PRGF from two donors for 6, 12, 24 and 48 h. Ki-67-gene expression was analyzed by real-time PCR. Shown are means ± s.e.m of three stimulations (** *p* < 0.01, *** *p* < 0.001; ns = non-significant, ANOVA with Bonferroni’s multiple comparisons test).

Furthermore, we asked if the PRGF treatment caused proliferation of primary human fibroblasts. To answer this question, we analyzed Ki-67 gene expression after stimulation of the fibroblasts with PRGF from five different donors (PRGF #1- PRGF #5, Figure 6A,B). These experiments revealed a significant Ki-67 gene induction in the fibroblasts after 24 h of PRGF stimulation (Figure 6B). A time-kinetic analysis of the PRGF-mediated Ki-67 gene induction in PRGF-treated fibroblasts revealed significant gene inductions after 24 and 48 h (Figure 6C).

**Figure 7:**
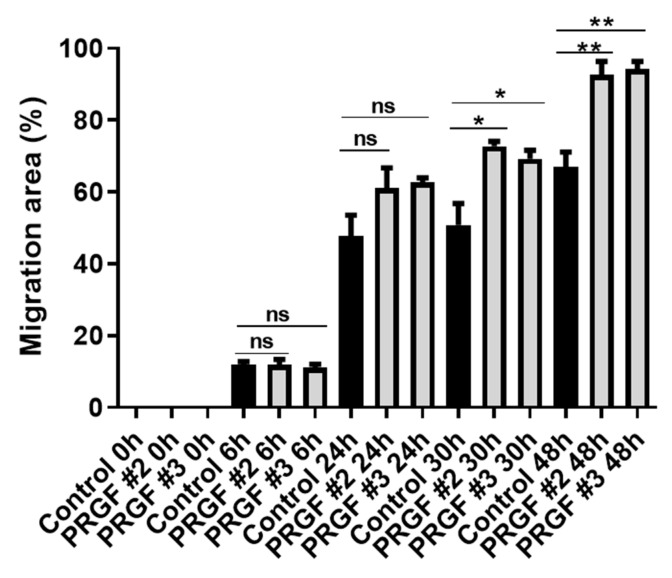
**PRGF enhanced migration of human primary fibroblasts in scratch assays**. Cultured human fibroblasts were Scheme 6. 24, 30 and 48 h. (*n* = 3, * *p* < 0.05, ** *p* < 0.01, ns = non-significant, ANOVA with Bonferroni’s multiple comparisons test).

To determine whether PRGF leads to an increased cell migration of fibroblasts, a scratch assay was performed. After inserting a gap in a confluent layer of cultured human fibroblasts by a pipette tip, the subsequent gap closure was monitored over time. This revealed a significantly faster gap closure by PRGF treatment after 30 h and 48 h incubation time (Figure 7).

## 3. Discussion

Thrombocyte products as Platelet-Rich Fibrin (PRF) or Platelet-released growth factors (PRGF) have been proven to be effective for the treatment of chronic or complicated wounds [3,4,11]. Underlying mechanisms are still insufficiently investigated. Data on the influence of thrombocyte concentrate products on the ECM physiology are especially rare. Recently, we have shown that PRGF induced several factors in primary human keratinocytes that play a role in ECM formation and we speculated that this might be one reason for the beneficial wound healing properties of thrombocyte concentrate products [10]. In the present study, we demonstrate that PRGF also induces several ECM-related factors in primary human fibroblasts. As fibroblasts are one of the major cellular players responsible for ECM formation, PRGF may strengthen ECM-formation also by its capacity to enhance the expression of ECM-associated factors in fibroblasts. In turn, this may contribute to the wound healing properties of thrombocytes-derived products [3,4,11,12]. In this study, we focused on nine factors that have been identified by whole transcriptome sequencing to be induced in PRGF-treated fibroblasts and which are all associated with ECM physiology. In the following, we will separately discuss these factors in more detail.

### 3.1. TGFBI

Transforming growth factor beta-induced protein (TGFBI) is an extracellular matrix protein secreted by several cells [13,14,15,16,17,18,19,20,21,22,23,24,25] that influences keratinocyte function [14], plays an essential role in extracellular matrix physiology [16] and increases the adhesion, migration and proliferation of epithelial cells [17]. A decreased TGFBi expression in fibroblasts was detected in chronic wounds [18], which supports the potentially important role of TGFBi in skin wound healing [18,19,20]. Thus, the observed PRGF-mediated induction of TGFBI in fibroblasts may contribute to the beneficial effects of thrombocytes-derived factors to support wound healing.

### 3.2. FN1

Fibronectin 1 (FN1) is an extracellular matrix molecule produced by various cell types, including fibroblasts and keratinocytes, that builds a bridge between cell surface receptors as integrins or collagens and other focal adhesion molecules. It plays an important role in the ECM synthesis and formation and regulates cell adhesion and migration [21,22,23]. FN1 promotes opsonization of tissue debris as well as migration, proliferation and contraction of cells involved in the complex processes of angiogenesis and wound healing [22,24]. Taken together, FN1 plays a crucial role in supporting epidermal injury repair processes [25,26,27,28,29,30,31,32]. Stimulation of the fibroblasts with PRGF caused the highest FN1 gene induction after 48 h, suggesting that an indirect paracrine or autocrine mechanism may be responsible for the observed induction. Accordingly, EGFR was not required for FN1 induction, suggesting that a direct activation by EGFR ligands plays no role in this context.

### 3.3. MMP9

MMP9 (matrix metalloproteinase 9) is a protease secreted by several cell types (e.g., fibroblasts) that is involved in many physiological processes including remodeling of the ECM. It degrades ECM proteins such as gelatin, collagen and elastin [33] and is essential for the removal of the fibrinogen matrix [34]. Furthermore, it is involved in keratinocyte migration and granulation tissue remodeling [35] and displays a key tissue remodeling enzyme that is indispensable for wound healing [36]. PRGF stimulation of primary human fibroblasts led to a significant MMP9 gene induction; after six hours, it was mediated by the EGFR. This suggests a direct activation of MMP9 by EGFR ligands present in the PRGF, a hypothesis that remains to be proven. It is noteworthy that we observed huge differences in the relative induction level of MMP9 in different experiments. This may be related to the fact that the MMP9 inducing EGFR ligands are highly donor-dependent, which is not the case for the other factors where EGFR activation is not necessary for induction by PRGF.

### 3.4. TGM2

TGM2 (transglutaminase 2) is a multifunctional cross-linking enzyme [37] that is involved in many biological processes in the human body [38,39], including the complex process of wound healing [39,40,41,42]. TGM2 causes tissue’s resistance to proteolytic degradation and enhances its’ mechanical strength [43]. In this context, it is involved in ECM stabilization by mediating the interaction of integrins with fibronectin [44]. In general, it is supposed to enhance wound healing and angiogenesis [38,45]. The TGM2 gene expression in fibroblasts was induced by PRGF after only 6 h, indicating a direct activation of expression by factors present in the PRGF. However, EGFR ligands seem to play no role as stimuli since blocking the EGFR by cetuximab did not decrease but rather increased the PRGF-mediated TGM2 induction. Thus, activation of the EGFR by PRGF may dampen the induction of TGM2 in this context.

### 3.5. FERMT1

FERMT1 (fermitin family member 1 or kindlin-1) is a focal adhesion protein that is involved in the assembly of the extracellular matrix (ECM) and re-epithelialization during wound healing as well as in the survival, proliferation, and differentiation of participating cells [46,47]. It plays a major role in the activation of integrins [48]. A FERMT1 deficiency is associated with severe cutaneous diseases and intestinal epithelial dysfunction [49,50]. In our experiments, we observed a very early FERMT1 gene induction in PRGF treated fibroblasts after only 6 h of stimulation. This induction was not dependent on the EGFR. Similarly, as discussed above for TGM2, this suggests a direct stimulation of FERMT1 by PRGF-provided stimuli.

### 3.6. COL1A1

Collagens are abundantly expressed by fibroblasts and form the scaffold of the ECM. Collagen type I alpha 1 chain (COL1A1) is involved in the formation of type I collagen fibers and is a major constituent of the dermis [51]. It plays also a critical role in wound healing [52]. The high expression of COL1A1 by fibroblasts is also reflected in the high amounts detected in the fibroblast culture supernatant by ELISA, which accords with a recent study demonstrating PRGF-mediated secretion of collagen type I by skin fibroblasts [53]. The increased release of COL1A1 by PRGF-stimulated fibroblasts indicates the beneficial influence of PRGF on collagen synthesis.

### 3.7. ADAM19

ADAM19 is a metalloproteinase of the ADAM (A disintegrin and metalloproteinase) family. As an endoprotease, it cleaves and activates growth factors. In addition, it is implicated in ECM degradation and reconstruction [54]. However, an abnormal high expression of ADAM19 is also linked to inflammation [54]. This may be related to the capacity of ADAM19 to shed tumor necrosis factor (TNF)-alpha [55]. Thus, one may speculate that the observed PRGF-mediated induction of ADAM19 may have positive effects on wound healing by facilitating remodeling of the ECM and promoting inflammatory events, which are critical steps in wound healing.

### 3.8. SERPINE1

The *SERPINE1* gene encodes the plasminogen activator inhibitor 1 (PAI-1). PAI-1 is a serine protease inhibitor (serpin) and plays a major role as an inhibitor of the fibrinolytic system by inhibiting tissue plasminogen activator (tPA) and urokinase plasminogen activator (uPA) [56]. PA-I contributes to control the synthesis of the ECM and is induced upon wounding and has a profound influence on ECM remodeling by blocking proteolytical collagen degradation [57]. PA-I also facilitates the migration of keratinocytes during wound healing and promotes epidermal injury repair [58,59,60]. PAI-1 is abundantly expressed by fibroblasts and its gene induction by PRGF suggests a regulative effect of PRGF on ECM remodeling during wound healing.

### 3.9. LOXL3

Lysyl oxidase-like 3 (LOXL3) is an amine oxidase that is required for the crosslinking of collagen and elastin in the ECM [61]. This is mediated by catalyzing the post-translational oxidative deamination of peptidyl lysine residues in precursors of elastin and different types of collagens [62]. Interestingly, the blockade of the EGFR by cetuximab increased the PRGF-mediated LOXL3 induction, suggesting an inhibitory influence of EGFR activation on LOXL3 expression. The possible interplay between EGFR and LOXL3 warrants further investigation.

In summary, all of the investigated factors, which are induced in PRGF-treated fibroblasts, play a role in the formation and remodeling process of the ECM. ECM reorganization is a crucial step during wound healing [63,64] and the above-mentioned studies reflect the potential functional impact and importance of these factors for generation and homeostasis of the ECM. Thus, the induction of these factors through thrombocytes extracts may promote the wound healing process by exerting beneficial effects on formation of the ECM.

Thrombocyte concentrate products contain a variety of growth factors, cytokines, and chemokines [65,66,67]. As we have recently demonstrated, the induction of antimicrobial peptides [5,6] and several factors involved in the ECM formation [10] in keratinocytes are dependent on the EGFR; in this study, we asked if the EGFR influences also the induction of the analyzed factors in fibroblasts. Surprisingly, except for MMP-9, the PRGF-mediated induction of all investigated genes was not inhibited after blocking the EGFR and some factors were even higher induced. This is in contrast to keratinocytes, where the PRGF-mediated induction of FN1, TGM2 and FERMT1 was dependent on the EGFR [10] indicating functional differences of the EGFR in keratinocytes and fibroblasts.

A huge difference regarding the influence of PRGF on fibroblasts and keratinocytes was also observed in the expression of Ki-67. In contrast to keratinocytes, where we observed a PRGF-mediated inhibition of Ki-67 expression [9], fibroblasts stimulated with PRGF revealed an induced Ki-67 expression. This was accompanied by increased migration in a scratch assay. These data are in line with the reported effects of PRGF to promote skin fibroblast proliferation and migration [53,68]. Since proliferation and migration of fibroblasts is important for wound closure, promotion of these steps may likely underlie the beneficial effects of thrombocytes extracts on wound healing.

In summary, our data indicate that PRGF caused significant induction of several genes in primary human fibroblasts that are essential for ECM formation. PRGF also promotes the proliferation and migration of the fibroblasts. These PRGF-mediated effects on fibroblasts can be another reason for the beneficial healing effects of chronic or complicated wounds under therapy with thrombocyte concentrate products such as PRGF or PRF.

## 4. Material and Methods

### 4.1. Preparation of PRGF

We produced PRGF from supernatants of freshly donated human thrombocyte concentrates as described before [8]. Briefly, thrombocyte concentrates were transferred into falcon tubes and centrifuged for 10 min at 2000 *g*. After the removal of the supernatant the thrombocyte pellet was washed twice with a sodium citrate buffer (0.11 mM, pH 5.5) and centrifuged again for 10 min at 2000 *g*. Thereafter, we removed the supernatant and resuspended the thrombocytes in half the volume of the initial thrombocyte concentrate volume using PBS. These resuspended thrombocytes were stored on ice, lysed by ultrasound, and stored at −80 °C for 24 h. The next day, we thawed the suspension, repeated the ultrasound procedure, and stored the suspension again at −80 °C for 24 h. On the third day, we thawed the suspension again and centrifuged it for 1 min at 18,000 *g*. The supernatant, the PRGF, was then removed and cryoasservated at −20 °C.

### 4.2. Culture and Stimulation of Primary Human Fibroblasts

Waste skin explants from surgeries were used to isolate human primary fibroblasts. The use of waste skin was approved by the local ethics committee of the Medical Faculty, University of Kiel, Germany (D 414/09; D 442/16) in concordance with the Declaration of Helsinki guidelines. The obtained samples were washed with phosphate-buffered saline, cut into defined pieces (0.25 cm^2^) and transferred into a 50 mL centrifuge tube containing a pre-prepared solution of 1 mL 2.5% trypsin and 25 mL PBS. After overnight incubation at 4 °C, 20 mL Dulbecco’s Modified Eagle’s Medium (DMEM, ThermoFisher Scientific, Dreieich, Germany) containing 10% FCS was added to neutralize the trypsin. The dermis was then mechanically separated from the epidermis and placed skin-side up in 6-well cell culture plates, with each well containing 6 dermis pieces. DMEM medium supplemented with 10% FCS (Capricorn Scientific, Ebersdorfergrund, Germany) and 1% Pen/Strep (ThermoFisher Scientific, Dreieich, Germany) was added (2 mL per well) and replaced twice a week. Incubation was conducted at 37 °C with 5% CO_2._ The dermis pieces were removed after a week. The outgrown fibroblasts were split at a confluence of 70–90% and transferred into cell culture flasks (75 cm^2^) for further cultivation. For stimulation, fibroblasts were seeded in 12-well tissue culture plates (BD Biosciences, Franklin Lakes, NJ, USA) in RPMI. At 90–100% confluence, the fibroblasts were stimulated with PRGF (1:10 diluted in RPMI) for the indicated period. To analyze the influence of the epidermal growth factor receptor (EGFR), we used the EGFR-blocking antibody cetuximab (Merck, Darmstadt, Germany) at a concentration of 20 µg/mL.

### 4.3. Real-Time PCR

After stimulation, total RNA was isolated and reverse transcribed in cDNA as described [69]. The cDNA served as a template in a real-time PCR using a fluorescence-temperature cycler (StepOne Plus; ThermoFisher Scientific, Dreieich, Germany) as described [69]. PCR was conducted using an annealing temperature of 60 °C for all reactions and serial dilutions of cDNA were used to obtain gene-specific standard curves for relative quantification of gene expression. The expression levels of the indicated genes were adjusted to the expression of the house-keeping gene RPL38 (ribosomal protein L38). The sequences of the used intron-spanning primer are shown in Table 1.

### 4.4. Enzyme-Linked Immunosorbent Assay (ELISA) Analysis

The concentration of fibronectin 1 (FN1) and collagen type I alpha 1 (COL1A1) in the supernatants of PRGF-treated fibroblasts were determined by ELISA (R&D Systems, Minneapolis, MN; catalog no. DY1918-05 and DY6220-05). ELISA was performed according to the manufacturer’s protocol.

### 4.5. Scratch Assay

A scratch assay was performed with fibroblasts to investigate whether stimulation with PRGF leads to increased cell migration. Fibroblasts were cultured in a 12-well plate using DMEM (with 10% FCS, without antibiotics) until 90–100% confluence was reached. The wells were scratched once using a 100 µL pipette tip to generate a standardized gap in the cell layer. The cells were then left unstimulated or stimulated with 500 µL PRGF (1:10 diluted in DMEM) and closure of the gap was microscopically analyzed after 6, 24, 30 and 48 h and documented by microscopic images. An analysis of the pictures was conducted using AxioVision LE 4.2.8.0 software (Carl Zeiss Microscopy, Jena, Germany) by measuring the size of the gap where no cells were present. By comparing the size of the gap at different times of observation, the progress of the migration could be assessed.

### 4.6. Expression Analysis of ECM-Related Genes in Ex Vivo Skin Explants

Skin explants for ex vivo experiments were obtained as waste material from abdomen or breast reduction surgeries. This approach was approved by the local ethics committee of the Medical Faculty, University of Kiel, Germany (D 414/09; D 442/16). The obtained samples were washed with phosphate-buffered saline and cut into defined pieces (0.25 cm^2^). The samples were placed in reaction tubes filled with 240 µL DMEM without supplements together with 60 µL of PRGF and incubated at 37 °C in a humidified atmosphere with 5% CO2 for 24 h. Subsequently, RNA Isolation was performed with NucleoSpin RNA Kit (Macherey-Nagel, Düren, Germany), according to the manufacturer’s protocol. cDNA analysis was performed as described above.

### 4.7. Whole Transcriptome Sequencing (RNA-Seq)

Fibroblasts were stimulated with PRGF, and total RNA was isolated using the NucleoSpin RNA Kit (Macherey-Nagel, Düren, Germany) according to the manufacturer’s protocol. RNA libraries were prepared and sequenced on a hiSeq4000 (Illumina, San Diego, CA, USA) as described [10]. Raw mRNA sequencing data were processed using Cutadapt (version 1.15) to trim Illumina standard adapters, Tophat2 [70] (version 2.1.1) together with Bowtie 2 [71] (version 2.2.3) to map the reads to the human reference genome (GRCh38, Ensembl release 91), Samtools [72] (version 1.5) to clean and sort the mapped reads, and HTSeq [73] (version 0.10.0) to count the number of reads mapping to each gene. Genes were annotated according to the Gencode version 27 annotation gtf file. Differential expression analysis of stimulated vs. unstimulated fibroblasts was conducted using the DESeq2 [74] Bioconductor package (version 1.24.0). The analysis was performed using the parametric Wald test and independent filtering of the results. Differentially expressed genes were defined by a false discovery rate (FDR as defined by Benjamini-Hochberg) <5% and an absolute log2 fold change (LFC) >1 corresponding to a doubled or halved expression. Log fold change estimates were corrected using the DESeq2 inbuilt LFC shrinkage function with the *apeglm* [75] method. Gene enrichment analysis was performed using Clusterprofiler [76] Bioconductor package (version 3.12.0) for biological processes compiled from Gene Ontology [77].

### 4.8. Statistics

Statistical analyses and graphs were generated using GraphPad Prism 8 (GraphPad Software LLC, San Diego, CA, USA). Since the small sample size did not allow for reliable analysis of distribution of the data the non-parametric Mann-Whitney *U* test was used to analyze data shown in Figure 1, Figure 2B,C, Figure 5 and Figure 6B. Due to the small sample size, which does not allow for the use non-parametric tests, the other data where analyzed by Student’s *t*-test or ANOVA with Bonferroni’s multiple comparisons test (when more than one group was analyzed against an unstimulated control group, Figure 3, Figure 6C and Figure 7). A *p*-value < 0.05 was considered statistically significant.

## Figures and Tables

**Table 1:** Primer sequences used for gene expression analyses of the indicated ECM-related factors by real-time PCR.

Gene	Forward Primer	Reverse Primer
Transforming Growth Factor Beta Induced, TGFBI	ACCCAGAAGCCCTGAGAG	TGCAGCCCACCTCCAGTG
Fibronectin 1, FN1	ACAACGTCATAGTGGAGGCA	CATCCGTAGGTTGGTTCAAG
Matrix Metalloproteinase 9, MMP9	GACACGCACGACGTCTTCCA	CACTGCAGGATGTCATAGGTCA
Transglutaminase 2, TGM2	CTCAACCTGGAGCCTTTCTC	AGGGCCCGCACCTTGATGA
Fermitin Family Member 1, FERMT1	GATTCCAGTGACAACATGGAG	TCAAACTCGATGACCACCTG
Lysyl Oxidase Like 3, LOXL3	TACAGCGAGCTGGTGAATGG	CAGATGCGGCCTGTTCCA
A Disintegrin And Metallo-proteinase 19, ADAM19	GCAATGCCTCTAATTGTACCCTG	GAGCCAACAGCTTACACTGG
Serpin Family E Member 1, SERPINE1	CCTGGTTCTGCCCAAGTTCT	CGTGGAGAGGCTCTTGGT
Ki67	TGACTTCCTTCCATTCTGAAGAC	TGGGTCTGTTATTGATGAGCC
Ribosomal protein L38, RPL38	TCAAGGACTTCCTGCTCACA	AAAGGTATCTGCTGCATCGAA

## Supplementary Materials

The following are available online at https://www.mdpi.com/article/10.3390/ijms221910536/s1.
